# Age dependency of EQ-5D-Youth health states valuations on a visual analogue scale

**DOI:** 10.1186/s12955-020-01638-z

**Published:** 2020-12-12

**Authors:** Jim G. A. Retra, Brigitte A. B. Essers, Manuela A. Joore, Silvia M. A. A. Evers, Carmen D. Dirksen

**Affiliations:** 1 Department of Clinical Epidemiology and Medical Technology Assessment, Maastricht University Medical Centre, Maastricht, The Netherlands; 2 Care and Public Health Research Institute (CAPHRI), Maastricht University, Maastricht, The Netherlands; 3 Trimbos Institute, Netherlands Institute for Mental Health and Addiction, Utrecht, The Netherlands

**Keywords:** EQ-5D-Y, Health state valuation, Child, Adolescent

## Abstract

**Background:**

Examine whether the use of different ages has an impact on the valuation of EQ-5D-Y health states for a hypothetical child or adolescent.

**Methods:**

A survey was administered during regular classes among a convenience sample of university students in the Netherlands. Respondents first valued 6 EQ-5D-Y health states (2 mild, 2 moderate, 2 severe) describing a hypothetical child/adolescent of a certain age on a visual analogue scale (VAS). After 1 h respondents valued the same six health states again but this time the age of the child was different. Age differed between 4, 10 and 16 year old.

**Results:**

Number of respondents was 311. No significant differences in valuation of the six health states were found between the age of 10 and 16. One moderate health state was valued significantly better for a 4-year old compared to a 10 and a 16 year old. The same applied for one severe health state that was valued higher for a 4-year old compared to a 16-year old.

**Conclusion:**

Our study shows that, except for one moderate and one severe health state, other EQ-5D-Y health states were not valued significantly different when description of age differed. It is possible that problems in specific health domains are considered more severe for older children/adolescents compared to younger children who might still be dependent on their caregivers. Future research should examine whether our findings are also present in a broader set of EQ-5D-Y health states, with a choice-based method like TTO or DCE, and a more heterogeneous sample.

## Background

Over the last years, interest in the use of preference based outcomes for economic evaluations in the paediatric population has increased [1–3]. Despite this attention, there is still a lack of child specific values for health states. These values are necessary in order to calculate Quality Adjusted Life Years (QALY’s) in cost-utility analysis [4].

One of the most frequently used health related preference based instruments in adults is the Euroqol 5D (EQ-5D-3L) [5]. This instrument consist of a descriptive part with five domains i.e. mobility, self-care, usual activities, pain/discomfort and anxiety/depression. In the three level version, each domain has three answer options leading to 243 unique health states. A tariff for the adult version has been developed based on valuations given by the general population for a subset of these EQ-5D-3L health states [6]. As from 2009, a child-friendly version of the EQ-5D-3L is available which is called the EQ-5D-Y (youth). The EQ-5D-Y is designed for self-completion by children/adolescents aged 8–15 with some slightly revised dimensions and answering descriptions compared to the adult version (www.euroqol.org). The questionnaire proved to be a useful and reliable tool to measure health related quality of life in children-/adolescents age 8–18 [2]. The EQ-5D-Y, however, lacks a tariff which limits the use of the instrument in economic evaluations [1].

In order to develop a tariff for the EQ-5D-Y, there are some challenges. One is the choice for an appropriate source to value the EQ-5D-Y health states [1]. For the development of the EQ-5D-3L tariff, a representative sample of the general population was asked to imagine and value a hypothetical health state description from their own perspective [6]. Since adults of all different ages were represented in the sample, the given values are in principle applicable for every age. This approach might be more difficult for the EQ-5D-Y because it implies that a representative sample of children/adolescents between the age of 8 and 15 would have to value health states from their own perspective. However, research has indicated that young children are often cognitively and/or language wise not able to participate in health state valuation experiments [7]. It has also been suggested that it might not be ethical to confront children with death in a health state valuation experiment [8]. Recently, the international valuation protocol for the EQ-5D-Y-3L was published [9]. One of the key features is that given the abovementioned difficulties with valuing health states by children, the valuation of EQ-5D-Y health states will be obtained by a sample of the general population. In addition, the framing of the valuation task is focused on a 10-year old child [9]. Previous studies found different valuations for a health state depending on whether this state was attached to a child or an adult [10–12]. However, the question is whether valuations for health states are also different when it concerns children or adolescents, as this reflects a much smaller age range than in the above mentioned studies. This is also acknowledged in the international valuation protocol for the EQ-5D-Y-3L in which the authors describe that as preferences regarding the health of 10 year old children might differ from preferences regarding the health of children or adolescents of other ages, further research is needed on this topic [9].

Given that the impact of the description of a specific age within the EQ-5D-Y valuation is largely unknown, this study was conducted. The aim was to examine the influence of different age descriptions on scores of EQ-5D-Y health states by using the EQ-5D visual analogue scale (VAS) scale. Hence, it was not the objective to obtain values in the context of a tariff. The following research question was formulated: Is the valuation of EQ-5D-Y health states different depending on the description of the age of a child/adolescent?

## Methods

### Recruitment and the sample

A convenience sample was chosen among Bachelor and Master students of the Faculty of Health, Medicine and Life Sciences at the Maastricht University in the Netherlands. Respondents were approached via their teachers. The questionnaire was handed out in March and April 2017 during regular classes.

### Survey design

The questionnaire was a self-completion paper-and-pencil questionnaire which consisted of different parts. First, the objective of the study was explained. This means that it was clarified that the study examined the valuation of quality of life of children in general. Thus, it was not explained that the questionnaire included different ages or that the impact of age on the valuation of a health state was examined. Potential participants could decide whether they wanted to participate. Second, following their consent, respondents were asked to value their own health state on the EQ-5D VAS-scale. Then the actual experiment started which consisted of two parts. Before the start of their class, respondents first valued 6 health states (two mild, two moderate, two severe) describing a hypothetical child/adolescent of a certain age. After they valued the last health state, they were asked in the following page to stop, keep the questionnaire and fill in the second part at the end of their class. In the second part, respondents valued the same six health states again but this time the age of the child/adolescent was different. Six versions of the questionnaire that differed in ordering of age (4 and 10 years, 10 and 4 years, 4 and 16 years, 16 and 4 years, 10 and 16 years or 16 and 10 years) and the ordering of the health states (mild, moderate and severe) were randomly distributed. The different ages of 4, 10 and 16 years were chosen to reflect the different development phases from childhood into adolescence. In addition, the age of 10 year old for the description of a hypothetical child within a EQ-5D-Youth health state has previously been used by Kind et al. and Kreimeier et al. [11, 13]

Finally, background questions like gender, age, religion, siblings, parental experience, birthplace, birthplace parents and two questions about the ease of completion of the valuation task were asked.

### Valuation task design

Participants valued six hypothetical EQ-5D-Y health states with a duration of 1 year (Table 1). Similar to a previous study by Kind et al. [11], we decided to provide no further information beyond the period of 1 year. The states chosen for this study are based on the health states and their classification used for the development of the UK and Dutch EQ-5D-3L tariff [6, 14]. In order to capture a range of potential problems with a health state, one very mild, one mild, two moderate and two severe health states were used in the experiment. The “age” of the child/adolescent was mentioned in a separate bullet point under the health descriptions.

**Table 1:** Health states based on Dolan [6]

Severity	Health state digits	Health state description
Very mild	11121	No problems in walking about, no problems in washing or dressing, no problems in doing usual activities, some pain or discomfort, not worried, sad or unhappy
Mild	11312	No problems in walking about, no problems in washing or dressing, a lot of problems in doing usual activities, no pain or discomfort, a bit worried, sad or unhappy
Moderate	13311	No problems in walking about, a lot of problems in washing or dressing, a lot of problems in doing usual activities, no pain or discomfort, not worried, sad or unhappy
Moderate	22222	Some problems in walking about, some problems in washing or dressing, some problems in doing usual activities, some pain or discomfort, a bit worried, sad or unhappy
Severe	23232	Some problems in walking about, a lot of problems in washing or dressing, some problems in doing usual activities, a lot of pain or discomfort, a bit worried, sad or unhappy
Severe	33212	A lot of problems in walking about, a lot of problems in washing or dressing, some problems in doing usual activities, no pain or discomfort, a bit worried, sad or unhappy

The EQ-5D-Y health states were valued on the EQ-5D VAS, which is a 20 cm thermometer with defined endpoints varying from a score of 0 as “worst imaginable health” and “best imaginable health” with a score of 100. Respondents were asked to put a cross in this thermometer to indicate their valuation and write the corresponding score in a box left to the VAS scale. Each health state was presented with a corresponding VAS scale on a separate blank page.

### Data analysis

Data was analyzed with the computer program IBM SPSS 23. In order to test whether the health state valuation by respondents was statistically different depending on description of the age of the child, a linear mixed-effects regression model was used. The reason for using this model is that, that is variation in scores within a student (before and after regular class) and between respondents is taken into account.

## Results

### Participant characteristics

In total 311 students participated in this study. One respondent who valued all mild and moderate health states lower (i.e. worse) compared to the severe health states was excluded from the analysis. Hence, 310 respondents were used for the analysis. Table 2 shows the respondents characteristics. Seventy-six percent is female and the mean age of the participants is 22 years old. The reported average health status is 76.8 (SD: 11.5, Median: 80 IQR: 70–85) and 72% of the respondents reported a very good or excellent health status during their youth.

**Table 2:** Characteristics of the study sample

	N	%
Gender		
Male	75	24
Age		
18**–**20	156	51
21**–**28	130	43
29**–**51	18	6
Health status (VAS)	76.8	
Health status during youth		
Fair	10	3
Good	77	25
Very good	143	47
Excellent	76	25
Religion		
Christian	151	49
Atheist	111	36
Other	47	15
Country of birth		
The Netherlands	238	77
Rest of Europe	59	19
Asia	7	2
North America	3	1
Africa	2	1

### Valuation results

Table 3 presents the average values for the EQ-5D-Y health states. Results show that participants valued on average the 6 health states in a logical way. The mild health states 11121 and 11312 were valued higher than the moderate health states 13311 and 22222. The moderate health states were valued higher compared to the severe health states 33212 and 23232. The only exception is the moderate health state 13311 ascribed to a 4-year-old (59.2) which was on average valued slightly higher (i.e. better) compared to the mild health state 11312 (58.5).

**Table 3:** Average values on a visual analogue scale per age

Health state	Age for hypothetical child/adolescent	N	Mean	SD
11121 (very mild)	4	204	72.6	13.4
11312 (mild)	4	205	58.5	13.4
13311 (moderate)	4	204	59.2	15.8
22222 (moderate)	4	205	54.8	14.2
33212 (severe)	4	204	48.6	15.8
23232 (severe)	4	205	41.1	15.0
11121 (very mild)	10	206	73.0	11.4
11312 (mild)	10	206	57.3	12.9
13311 (moderate)	10	206	54.8	13.6
22222 (moderate)	10	206	54.0	13.2
33212 (severe)	10	206	46.4	13.9
23232 (severe)	10	206	39.4	14.7
11121 (very mild)	16	207	74.0	10.3
11312 (mild)	16	206	57.6	12.0
13311 (moderate)	16	206	56.0	13.5
22222 (moderate)	16	206	54.2	12.9
33212 (severe)	16	206	45.6	13.6
23232 (severe)	16	206	39.2	13.5

### Differences in valuation depending on age

Table 4 shows the results of the linear mixed effect regression model. No significant differences in valuation of the health states were found between the age of 10 and 16. For the comparison of a 4-year old versus a 10-year old, the moderate health state (13311) was valued significantly higher for a 4-year old. For the comparison of the age 4 versus 16, again, the moderate health state 13311 was valued significantly higher for a 4-year old compared to a 16-year old. In addition, the severe health state 33212 was valued higher, i.e. better, for a 4-year old compared to a 16-year old.

**Table 4:** Health state valuations between and within participants

	Mean difference	SE	95% CI	Sig
11121 (very mild)				
4 versus 10	− 0.375	0.98	− 2.3; 1.6	0.703
4 versus 16	− 1.695	0.98	− 3.6; 0.2	0.086
10 versus 16	− 1.320	0.98	− 3.2; 0.6	0.179
11312 (mild)				
4 versus 10	0.822	0.988	− 1.1; 2.8	0.406
4 versus 16	0.967	0.988	− 1; 2.9	0.328
10 versus 16	− 0.822	0.985	− 1.8; 2.1	0.882
13311 (moderate)				
4 versus 10	3.725	1.221	1.3; 6.1	0.002**
4 versus 16	3.809	1.221	1.4; 6.2	0.002**
10 versus 16	0.083	1.215	− 2.3; 2.5	0.945
22222 (moderate)				
4 versus 10	0.191	1.099	− 2; 2.4	0.862
4 versus 16	0.064	1.099	− 2.1; 2.2	0.954
10 versus 16	− 0.127	1.096	− 2.3; 2	0.907
33212 (severe)				
4 versus 10	1.274	1.133	− 1.3; 3.5	0.261
4 versus 16	2.887	1.132	0.7; 5.1	0.011**
10 versus 16	1.612	1.128	− 0.6; 3.8	0.154
23232 (severe)				
4 versus 10	0.738	1.182	− 1.6; 3	0.533
4 versus 16	1.527	1.181	− 0.8; 3.8	0.197
10 versus 16	0.789	1.176	− 1.5; 3.1	0.503

**Significant *P* < 0.05

### Ease of completion

The large majority (90.5%) of the respondents answered that the EQ-5D-Y descriptions of health states were clear enough and that no aspects were missing. The main reasons why 9.5% of the participants did not find the health states clear enough were related to (1) problems with differentiating between the answer options of a bit, some, a lot of, (2) thoughts about the health state descriptions being illogical and (3) the lack of background information about the child in the task such as the relationship with parents or social interactions.

## Discussion

The main objective of this study was to examine whether age ascribed to a child/adolescent in the description of a health state influenced values given by respondents. Results showed that, except for one moderate and one severe health state, other EQ-5D-Y health states were not valued statistically significantly different when the ascribed age differed. The statistically different values for the moderate health state 13311 occurred in the comparison 4-year old against 10 and 4-year old against 16 year. In both cases, this health state was valued higher for a 4-year old. The same holds true for the severe health state 33212 that was valued higher for a 4-year old compared to a 16-year old. As this is the first study exploring the impact on age on health state valuations *within* the child/adolescent age range (in our study 4–16 years), our results cannot be compared to other studies that have compared health state valuations in children compared to those in adults and used different methods [11–13].

An explanation for the higher values in the moderate and severe health state for a 4-year old can perhaps be found in the description of the EQ-5D-Y domains. The child in the moderate health state 13311 suffers from a lot of problems in washing or dressing and in usual activities. In the severe health state 33212 the child suffers from a lot of problems in walking, a lot of problems in washing or dressing, some problems in doing usual activities and is a bit sad or unhappy. The two health states have in common that the child/adolescent experiences severe problems with at least two of the ‘functioning’ domains. It is possible that in particular problems with these domains are considered more severe for older children/adolescents (10- and 16-year old) compared to younger children (4-year old) who are usually still dependent on their caregivers and thus not completely autonomous. Hence, it may be perceived as relatively less problematic in relation to the developmental stage of young children and not necessarily be caused by *health problems* only. One might argue that that the higher values for a 4-year old are no cause for concern, because the EQ-5D-Y is originally designed for self-completion by children and adolescents aged 8–15 years. Our finding is, however, relevant since a proxy version of the EQ-5D-Y is also available, which can be used for children as from the age of 4 (www.euroqol.org).

Perhaps an adapted proxy version of the EQ-5D-Y for (very) young children in which the selection/wording of domains and/or response options align better with their developmental stage, might be a way forward. Interestingly, the severe health state 23232 with a lot of problems in the domain pain/discomfort showed no difference between the age of 4, 10 or 16 year. It could be that with severe pain, a dimension directly related to health, people tend to have a similar judgement regardless of age. Still, the extent to which some health dimensions potentially can lead to differences in valuation of health between different ages and others not could be an area for further research.

For this explorative study, a convenience sample of young adult university students was used. A limitation of a convenience sample is that it is not representative of the general population. Especially in this case, the respondents were young highly educated adults. Hence, our findings cannot be generalized. The objective of this study, however, was to gain knowledge about the methodological issue of age-dependency in the valuation of EQ-5D-Y health states which made a representative sample of the general population not a necessity. Another limitation might be that we selected health states that have previously been used for the valuation of the UK and the Dutch tariff of the EQ-5D-3L [6, 14]. In hindsight, one could question whether a health state with severe problems in usual activities (11312) can really be considered mild. Perhaps this is also why some respondents considered a health state description illogical. Nevertheless, at the time of the valuation studies, this health state was described as mild and considered to be plausible for respondents [6]. Strengths of our study are the large sample size and the fact that we controlled for an ordering effect by changing the order of both age and health states. The method for deriving health states values for six EQ-5D-Y health states in our study was the EQ-5D VAS method. Although the VAS is an easy method to use, it is not an instrument that requires a trade-off between life years and quality of life which is considered necessary for the estimation of a tariff. Hence, further research should examine whether the results as found in this study are also present when applying a choice-based method like the Time Trade-Off (TTO) or a Discrete Choice Experiment (DCE). Earlier research concerning the valuation of EQ-5D-Y health states used the age of 10 in health state descriptions [11]. One could argue that this age might be a legitimate choice to use in the description of the EQ-5D-Y health states since the EQ-5D-Y questionnaire is designed for self-completion by children and adolescents aged 8–15 years (www.euroqol.org).

## Conclusion

Given the potential difficulties with valuing health states by children, the valuation of EQ-5D-Y health states could be performed by a sample of the general population. Such an approach, however, requires in general more information like age of the child /adolescent for respondents because they are asked to value a perspective that is different from their own. The aim of our study was to examine the influence of different age descriptions on scores of EQ-5D-Y health states by using the EQ-5D visual analogue scale (VAS) scale. Our research has shown that, except for one moderate and one severe health state, other EQ-5D-Y health states were not valued statistically significantly different when the ascribed age differed. However, future research should examine whether our findings are also present in a broader set of EQ-5D-Y health states, with a choice-based method like TTO or DCE and a more heterogeneous sample.

## Data Availability

The datasets used and/or analyzed during the current study are available from the corresponding author on reasonable request.

## Abbreviations

VAS: Visual analogue scale
EQ-5D-Y: Euroqol-5 dimensions-youth
TTO: Time trade off
DCE: Discrete choice experiment
QALY: Quality adjusted life year
